# Trends in thyroid hormone prescribing and consumption in the UK

**DOI:** 10.1186/1471-2458-9-132

**Published:** 2009-05-11

**Authors:** Anna L Mitchell, Bryan Hickey, Janis L Hickey, Simon HS Pearce

**Affiliations:** 1 Endocrine Unit, Royal Victoria Infirmary and Institute of Human Genetics, Newcastle University, Newcastle upon Tyne, NE1 4LP, UK; 2 British Thyroid Foundation, 3 Devonshire Place, Harrogate, HG1 4AA, UK

## Abstract

**Background:**

Thyroid hormone replacement is one of the most commonly prescribed and cost effective treatments for a chronic disease. There have been recent changes in community prescribing policies in many areas of the UK that have changed patient access to necessary medications. This study aimed to provide a picture of thyroid hormone usage in the UK and to survey patient opinion about current community prescribing policies for levothyroxine.

**Methods:**

Data on community prescriptions for thyroid hormones in England between 1998 and 2007, provided by the Department of Health, were collated and analysed. A survey of UK members of a patient support organisation (the British Thyroid Foundation) who were taking levothyroxine was carried out.

**Results:**

The amount of prescribed thyroid hormones used in England has more than doubled, from 7 to almost 19 million prescriptions, over the last 10 years. The duration of prescriptions has reduced from 60 to 45 days, on average over the same time. Two thousand five hundred and fifty one responses to the patient survey were received. Thirty eight percent of levothyroxine users reported receiving prescriptions of 28 days' duration. 59% of respondents reported being dissatisfied with 28-day prescribing.

**Conclusion:**

Amongst users of levothyroxine, there is widespread patient dissatisfaction with 28-day prescription duration. Analysis of the full costs of 28-day dispensing balanced against the potential savings of reduced wastage of thyroid medications, suggests that this is unlikely to be an economically effective public health policy.

## Background

Since 1968, most forms of hormone replacement therapy, including levothyroxine, have been dispensed free of charge to NHS patients in England and Wales, under the medical exemption scheme. Thus, patients taking essential medications have been able to access a continuous supply, irrespective of their means. Patients with both autoimmune and congenital hypothyroidism require thyroid hormone replacement, in addition to those who have had surgical thyroidectomy or ablative radioiodine treatment for hyperthyroidism and thyroid cancer. Since hypothyroidism from all these causes is a chronic and irreversible condition, the majority of hypothyroid patients will require lifelong thyroid hormone treatment. About 19 million prescriptions for thyroid hormone preparations were dispensed in England during 2007, making it one of the most frequently prescribed medications[1]. From the above data, one can estimate that slightly over 3% of the population of England were prescribed regular levothyroxine during 2007[2]. This is corroborated by a prevalence rate for hypothyroidism of 3.01% in Tayside, Scotland during 2001[3].

Hypothyroidism as a clinical syndrome was first recognised in the 1870s and its subsequent treatment with extract of animal thyroid was first achieved in Newcastle upon Tyne during the 1890s by Murray[4]. Synthetic thyroid hormone replacement therapy has been available since 1927, when British chemists Harington and Barger first synthesised thyroxine[5]. So, for more than 50 years, thyroid hormone replacement has predominantly been formulated as synthetic levothyroxine (T4). Nevertheless, in recent years there has been a minor trend away from levothyroxine monotherapy in the treatment of hypothyroidism. This has taken two distinct forms: use of tri-iodothyronine (liothyronine, T3) either as monotherapy, or more commonly combined with levothyroxine (T3/T4); and use of desiccated porcine thyroid (marketed as Armour 'Natural' thyroid). The use of combined T3/T4 was re-explored following a small but high profile study of thyroid cancer patients who were swapped from suppressive levothyroxine therapy, to a lesser dose of combined T3/T4[6]. Subsequent to this study, a further 10 larger studies, involving, in total, more than 1000 patients largely with autoimmune hypothyroidism, have failed to reproduce a benefit from combined T3/T4[7]. Nevertheless, there is no current formulation of T3/T4 that replicates the natural pattern and relative quantities of these hormones released from the human thyroid, and a slow release preparation might have utility in the future. The movement towards use of porcine thyroid extract in the UK has been largely patient-led, with the support of a few fringe practitioners, with many patients believing there could be some additional benefit from use of a 'natural' preparation compared to use of synthetic hormones. As there has never been a randomised trial of levothyroxine versus porcine thyroid extract, any possible health benefit remains uncharacterised, although most conventional practitioners have been cautious to recommend such therapy, as porcine thyroid is known to synthesise substantially more T3 than human thyroid[8]. In addition, porcine thyroid extract is substantially more expensive than the 4 pence for a 100 microgram levothyroxine tablet.

As concerns grow over increasing healthcare costs, local primary care organisations (PCOs) have developed strategies aimed at rationalising resource use and providing good value in health care spending. Prescription drug wastage and over-prescribing have been identified as particular targets for this economy drive, and over the last 5 years many PCOs have implemented new initiatives to reduce drug costs. One such strategy has been for PCOs to recommend that GPs prescribe only a 28-day supply of medication at one time. This 28-day prescribing policy followed several studies which demonstrated that restricted and closely monitored prescribing periods reduced over-prescribing and medicine wastage[9-11]. One scheme introduced in Kirklees estimated that by doing this, drug wastage would be reduced by approximately 33%[11]. Nevertheless, most PCOs have recognised that for certain medications, most notably oral contraceptives, the detrimental effect of an interrupted patient supply would not be acceptable, and therefore exempted these from the policy. In a similar way, several PCOs have seen that for inexpensive and long-term medications (eg. oestrogen hormone replacement), there are few savings to be made and have exempted these from the 28-day prescribing policy. However, many practices in the UK have applied the 28-day prescription recommendation indiscriminately and without flexibility, and this may have had an untoward effect on numerous patients taking long-term medications, including levothyroxine. The impact of this prescribing policy on, and its acceptability with, patients taking regular medications has never been evaluated. In this paper, we document the trends in prescription of thyroid hormones in England over the last 10 years. We also provide the first information on the implementation of the 28-day prescribing recommendation across the UK with relation to levothyroxine, including patient perceptions and satisfaction with the policy.

## Methods

### Trends in thyroid hormone prescribing; England 1998–2007

Trends in prescription drug usage were monitored by analysing annual data published by the Department of Health (DoH), known as Prescription Cost Analysis (PCA) statistics. The PCA provides details of the number of items and the net ingredient cost of the prescriptions dispensed from community pharmacies in England. These data are based on information obtained from prescriptions sent to the Prescription Pricing Division for payment and include all prescriptions dispensed in the community. This includes prescriptions dispensed by pharmacists, appliance contractors, dentists, general practitioners, prescriptions from hospital doctors dispensed in the community and items personally administered by doctors. We analysed the PCA data for England only, which are released on a yearly basis, from April 1998 to 2007, inclusive. By analysing PCA data, it is possible to ascertain the trends in thyroid hormone usage on a year by year basis and therefore the cost of thyroid hormone usage to the NHS. Data extracted included the number of thyroid hormone prescriptions dispensed in England per year, the cost of these prescription items and data to allow the calculation of the approximate prescription length administered (quantity of tablets per prescription). Information about dispensing tariffs was obtained from the prescription pricing division of the NHS business services agency.

### Questionnaire survey of British Thyroid Foundation (BTF) membership 2007

To explore the perceptions of the 28-day prescribing recommendation amongst patients receiving thyroid hormone replacement treatment, a questionnaire was designed by the authors and reviewed by a patient members panel of the British Thyroid Foundation (BTF). This was distributed by the BTF with their quarterly newsletter in August 2007. The BTF is a registered charity that works with medical professionals to provide information and support to people with thyroid disorders. The quarterly newsletter was distributed to 6121 members of the BTF, of whom 6083 were UK residents. The survey aimed to explore how long respondents had been taking thyroid replacement treatment, how they obtained their prescriptions, and their concerns, comments and experiences about 28-day prescribing. The full questionnaire is available on request from the authors (see additional file 1). Ethics committee approval was not needed for this study as it was instigated at the request of BTF representatives and not performed on any NHS site. Permission to use anonymous data for the purposes of publication was granted by the Trustees of the BTF.

## Results

### Thyroid hormone usage in England

**Figure 1:**
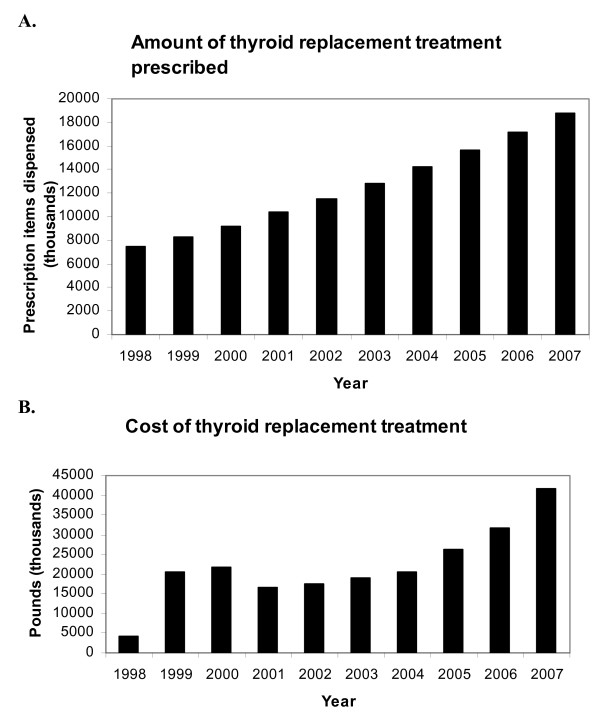
**Quantity and cost of thyroid hormone replacement in England**. **Panel A **– The number of prescription items dispensed in thousands for all thyroid hormone replacement therapies (levothyroxine, liothyronine and Armour thyroid) from 1998 to 2007 (PCA data). **Panel B **– Net ingredient cost of thyroid hormone replacement therapy in England from 1998 to 2007.

Since 1998, thyroid hormone usage, and therefore the cost that these prescriptions incur, has increased dramatically. The number of prescription items dispensed in England for all forms of thyroid hormone replacement has more than doubled from just over 7 million in 1998 to close to 19 million in 2007 (figure 1, panel A). Levothyroxine (T4) is the most commonly prescribed thyroid hormone, representing over 99.7% of all thyroid hormone prescriptions. Liothyronine (tri-iodothyronine, T3) and Armour thyroid make up the rest of thyroid hormone prescriptions, totalling 0.30 and 0.008%, respectively in 2007. Figure 1, panel B illustrates the total yearly cost of thyroid hormone replacement therapy prescriptions.

**Figure 2:**
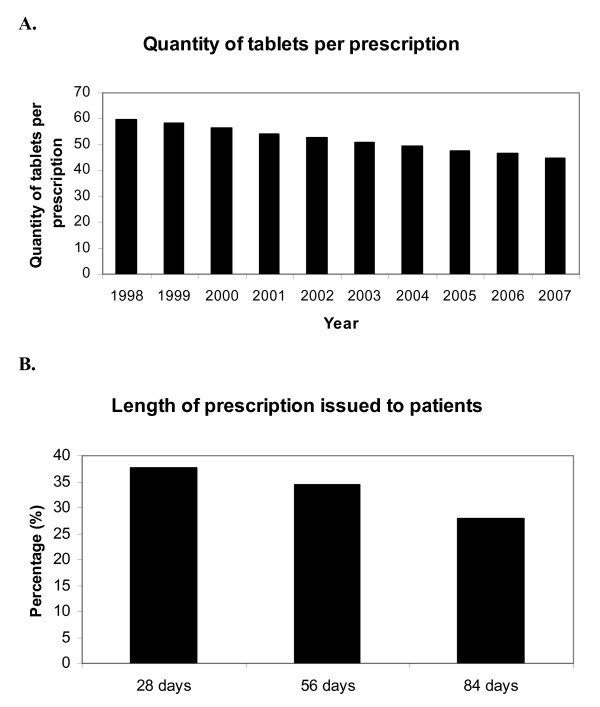
**Length of levothyroxine prescriptions**. **Panel A **– Temporal trend in the mean prescription content, for levothyroxine 100 μg tablets, from 1998 to 2007 in England (PCA data). **Panel B **– Length of prescription issued to patients responding to BTF survey (n = 2551 responses).

The PCA data also reveal a change in the content of prescriptions over the 10-year period of observation (figure 2, panel A). During 1998, there were just over 2.8 million prescriptions for thyroxine 100 μg tablets in England and the mean content of each prescription was 59.7 tablets. This is sufficiently close to the figure of 56 tablets to assume that the mode prescription length was likely to be 56-days or 8 weeks. By 2007, there were 7.0 million prescriptions for levothyroxine 100 μg tablets and the mean content of each prescription was reduced to 44.9 days, slightly over 6 weeks.

### British Thyroid Foundation member survey

Two thousand, five hundred and fifty one individuals taking levothyroxine responded to the questionnaire (42% of those dispatched to UK residents), which enquired about patient experiences of obtaining levothyroxine and sought opinions about restricted prescription lengths. Ninety six percent of respondents had been taking levothyroxine tablets for more than a year, with more than 70% of people taking them for more than 5 years. With regard to length of prescription available, 38% of respondents were being prescribed levothyroxine for 28 days at a time, with only 28% receiving prescriptions for 3 months (84 days); figure 2, panel B.

**Figure 3:**
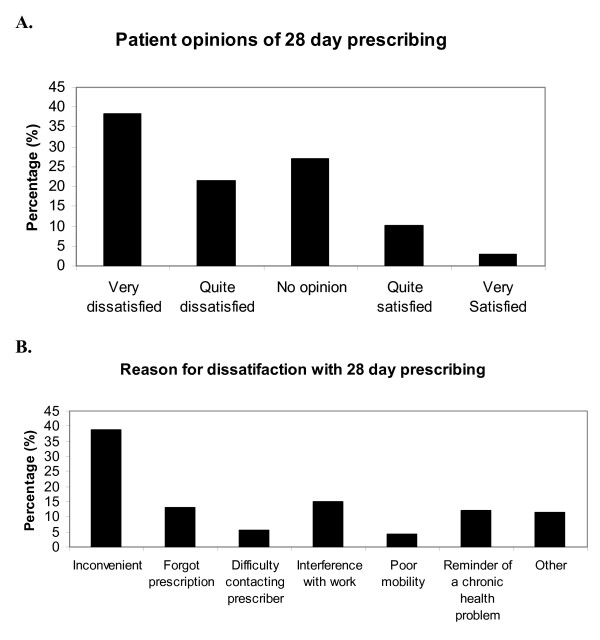
**Patient opinion about 28-day prescribing**. Data taken from BTF survey responses. **Panel A **– Patient satisfaction with the 28 day prescribing policy. **Panel B **– Most frequent responses of reasons for dissatisfaction with the 28 day prescribing.

The majority of respondents describe being unhappy with the 28-day prescribing arrangement, 59% of people being dissatisfied overall, compared to just 13% feeling satisfied (figure 3, panel A). However, fewer than half of people who were prescribed levothyroxine for 28 days had asked their primary care practitioners for a longer prescription. Of those who had asked, it appears that approximately half of practitioners had agreed to prescribe a longer amount, with the other half declining to extend the prescription when asked. Seventeen percent of people responding to the survey admitted to having missed levothyroxine tablets, with 6% having gone without tablets on more than one occasion, owing to a lack of dispensed medication.

When asked what the major reasons for dissatisfaction with the 28-day prescribing policy were, the two most common responses were that it is simply inconvenient to pick up the prescription/tablets more frequently or that it interferes with the working day (figure 3, panel B). In free text comments several other common themes emerged:

• In rural areas people have to travel significant distances to get their prescription, sometimes making more than one journey per prescription (e.g. to order it, pick it up and have it dispensed).

• Those with physical disabilities described problems getting to the practice.

• Many people feel that asking for a repeat prescription is a waste of their doctor's time and that they don't want to bother the doctor for something so simple.

• Working long hours or shift-work make it difficult for many people to get to the surgery or pharmacy during limited opening times.

• Some people leave the UK on holiday for more than a month each year, and this also causes a problem.

• In contrast, when other medications are being prescribed every 28 days, several people comment that it makes it easy to remember that all the prescriptions are for 28 days, and that they have to go back for their other medications anyway.

### Analysis of non-drug costs

Levothyroxine is a remarkably cheap drug, costing about £1.12 for a 28-day supply of 100 μg tablets; the total yearly cost for the drug itself being around £14.50 per patient at this dose. Thus, the professional fee for the pharmacist of £0.90 per item dispensed makes up a significant cost in the delivery of the treatment to the patient. These figures allow one to calculate that to treat a patient for a year with levothyroxine dispensed every 84 days costs around £18.40, whereas the professional fees to dispense the drug an additional 9 times during the course of a year of treatment, as required with a 28-day supply, incurs a total cost of £26.30. This estimate does not factor in additional costs incurred, such as practice receptionist or doctor time to print out, check and sign the additional prescriptions. Thus, 28-day prescribing of levothyroxine is substantially more expensive to the NHS than giving longer supplies. The increased costs of restricted prescription lengths is supported by work carried out in America which calculated that the cost of a variety of drug therapies actually increases with a shorter prescription length, 34 days, compared to a longer prescription of 100 days[12].

## Discussion

Analysis of the PCA data clearly shows an increase in the prescribing of thyroid hormone treatments from 1998 to 2007. There may be a number of possible explanations for this trend. Firstly, hypothyroidism becomes more prevalent with advanced age. It is likely that as longevity increases, increasing numbers of elderly people will require continued thyroid hormone replacement. Another contributory factor may be an increase in thyroid function testing by GPs. This may result in more patients with hypothyroidism being identified, or, perhaps more likely, earlier detection and treatment of individuals with subclinical hypothyroidism who may not previously have been identified or, had they been identified, started on treatment. With the recognition that radioiodine is a safe, acceptable and highly cost-effective treatment for women of childbearing age with thyrotoxicosis[13], it is possible that more patients with hyperthyroidism are rendered hypothyroid following radioiodine ablation at an earlier stage of their treatment. A final possibility is that public perceptions of health have shifted slightly, such that health is perceived as being more than simply the absence of disease, leading more people to aspire towards a positive sense of wellbeing. With this objective, more individuals are seeking "check ups" that include thyroid function testing in 'well person' clinics. Such people may then be found to have mild or subclinical hypothyroidism and be commenced on thyroid hormone treatment. Thus, it seems likely that a combination of the above factors has led to the dramatic rise in thyroid hormone usage.

The cost of thyroid hormone treatment to the NHS has also been increasing, in keeping with the trend of increased prescribing of thyroid hormone treatment. Even so, thyroid hormone treatment remains inexpensive compared with many drugs available, with 100 μg of levothyroxine costing just less than 4 pence.

Drug wastage seems to occur because many medical problems improve and subsequently resolve, at which point the patient then stops taking the medication. If a three-month prescription is issued in this instance, far more medication is wasted than if a one-month prescription is issued. Another reason for wastage of prescription drugs is that a person may stop taking them due to unwanted side effects, which are likely to occur soon after the drug is started. Restricting prescriptions to 28 days has been proposed to reduce drug wastage. The reported benefits[14] of the 28-day prescribing policy include:

1) Reducing confusion in patients by limiting the number of medication packets that they will have at home at any time.

2) Allowing medication to be made into a 28-day "blister pack" or dosing cassette making it easier for patients to remember to take their medications.

3) Having a fixed prescription period allows all the medications for a single patient to be dispensed and therefore renewed at the same time, thus reducing the number of prescriptions a practice has to process.

4) Having a fixed prescription period makes it easier for health care professionals to identify appropriate times for patients to have a review of their medication.

5) Having a fixed prescription period allows patients to have regular contact with a primary healthcare professional (doctor, nurse or pharmacist) so that any problems with their medications can be discussed and rectified.

6) A fixed prescription period would allow concordance issues to be more readily identified, as patients ordering prescriptions early or late can be easily identified.

Although the 28-day prescribing policy has some benefits in principle, in practice the policy has some clear disadvantages for certain groups of patients. This is particularly so for individuals taking inexpensive medication, lifelong. The analysis of the non-drug costs associated with levothyroxine usage (i.e. pharmacist's professional fees for dispensing, doctor and practice staff time) show that there is little hope of recouping cost for any wasted drug using a 28-day prescribing approach. We previously estimated that the extra professional dispensing fees for levothyroxine prescriptions alone would add at least £7 m of treatment costs annually in England[15].

Although the responses to the survey distributed to BTF members clearly represent only a proportion of national opinion, of those patients that responded, more than one third were currently being given 28-day levothyroxine prescriptions, and the majority of respondents were dissatisfied with this. There are several good reasons why this should be so (see free text comments above). In addition, more than one in six patients report having gone without their prescription medication due to difficulty in obtaining it. Furthermore, patients taking levothyroxine whose thyroid status is fluctuating or whose dose needs adjusting are generally recommended to alter the dose and have a repeat blood test in 8 to 12 weeks[2]. So the arbitrary nature of the 28-day recommendation makes little sense in terms of clinical practice either.

The PCA dispensing data was collected by the Prescription Pricing Division in a standardised way during the period of study, so we believe this data to be reliable. The British Thyroid Foundation survey was sent to 6083 UK members, although not all of them would have been levothyroxine users. While we do not know the exact response rate in people being prescribed levothyroxine, it is clearly a possibility that we have obtained the views only of the most disgruntled members; patients with no issues being less likely to respond. Thus, our analysis could be unrepresentative of the general opinion of levothyroxine users. Despite these possible limitations, there is no other published information of this nature available, and even if considered at a qualitative level only, the survey findings do provide an authentic insight into the patient experience.

## Conclusion

The data we present illustrate that a fuller and more considered analysis of 28-day prescribing recommendations should be carried out. Importantly, the likely impact of restricted prescribing duration on patients, balanced against the likelihood of cost saving, should be evaluated on a drug-by-drug basis. We feel that an indiscriminately applied 28-day prescribing policy is clearly inappropriate for cheap medications and for those medications that have to be taken lifelong. Our analysis may be equally applicable to several other categories of medications including those for other endocrine conditions (e.g. hydrocortisone[16]) and for the treatment of several other chronic conditions.

## Competing interests

JLH works for the British Thyroid Association which is a patient support group.

## Authors' contributions

JLH came up with the original concept for the paper, was involved in the BTF questionnaire design and contributed to revisions of the manuscript. BH analysed the BTF questionnaire data and contributed to revisions of the manuscript. ALM analysed the BTF questionnaire data, wrote the first draft of the manuscript and contributed to subsequent revisions. SHSP was involved in the BTF questionnaire design and contributed to revisions of the manuscript. All authors read and approved the final manuscript.

## Pre-publication history

The pre-publication history for this paper can be accessed here:



## Supplementary Material

Additional file 1 **BTF 28-Day Survey**. Survey distributed to BTF members to ascertain opinions of the 28-day prescribing policy. Click here for file
