# Supplementary material for: Trends in thyroid hormone prescribing and consumption in the UK
Source: BMC Public Health. 2009 May 11;9:132. doi: 10.1186/1471-2458-9-132 (PMC2683823; doi:10.1186/1471-2458-9-132)
Supplement: Additional file 1 — BTF 28-Day Survey. Survey distributed to BTF members to ascertain opinions of the 28-day prescribing policy. [file 1471-2458-9-132-S1.doc]

**Additional file 1**

**BTF 28 - Day Survey**

*Please answer all questions*

1. How long have you been taking levothyroxine tablets?

Less than 1 year ⬜ 1 to 5 years ⬜ 5 to 10 years ⬜ More than 10 years ⬜

2. How much levothyroxine are you routinely prescribed?

28 days ⬜ 56 days ⬜ 84 days ⬜ other ⬜.........(days)

3. If you are prescribed more than 28 days, did you have to ask your GP for a supply greater than 28 days?

Yes ⬜ No ⬜ Not applicable ⬜

4. If you are prescribed 28 days, have you ever asked your GP for a prescription for more than 28 days

Yes ⬜ No ⬜ Not applicable ⬜

5. If your answer to Question 4 is “Yes”, did your GP agree to prescribe more than 28 days?

Yes ⬜ No ⬜ Not applicable ⬜

6. If the answer to Question 5 is “No” what was the reason given?

PCT directive ⬜ Surgery Policy ⬜ No reason given ⬜ Other ⬜...........................................

7. How do you feel about 28 day prescriptions?

Very dissatisfied ⬜ Quite dissatisfied ⬜ No opinion ⬜ Quite satisfied ⬜ Very satisfied ⬜

8. If you are very dissatisfied or quite dissatisfied with 28-day prescriptions, what are the reasons?

(Tick all boxes that apply):

a. Inconvenience / waste of my time ⬜

b. I forget to ring ahead and book my prescription ⬜

c. It is difficult to contact the surgery ⬜

d. Going to the surgery interferes with working day ⬜

e. Problems with mobility make it difficult to pick up ⬜

f. I am constantly reminded that I have a health problem ⬜

g. Other (please state).................................................................................... ⬜

9. Have you ever gone without levothyroxine due to difficulties obtaining a prescription?

No, never ⬜ Yes, once ⬜ Yes, several times ⬜ Yes, most months ⬜

10. In what town do you live? ..................................................................Postcode .......................

11. Name of your Primary Care Trust (PCT) (if known) .............................................................................................

Thank you for completing the questionnaire. Please return it by 30th November 2007 to:

“28-Day Survey”, British Thyroid Foundation, PO Box 97, Clifford, Wetherby , West Yorkshire LS23 6XD
